# NONAN GaitPrint: An IMU gait database of healthy young adults

**DOI:** 10.1038/s41597-023-02704-z

**Published:** 2023-12-05

**Authors:** Tyler M. Wiles, Madhur Mangalam, Joel H. Sommerfeld, Seung Kyeom Kim, Kolby J. Brink, Anaelle Emeline Charles, Alli Grunkemeyer, Marilena Kalaitzi Manifrenti, Spyridon Mastorakis, Nick Stergiou, Aaron D. Likens

**Affiliations:** 1 Division of Biomechanics and Research Development, Department of Biomechanics, and Center for Research in Human Movement Variability, University of Nebraska at Omaha, Omaha, NE 68182 USA; 2 College of Information Science and Technology, University of Nebraska at Omaha, Omaha, NE 68182 USA; 3 Department of Physical Education and Sport Science, Aristotle University, Thessaloniki, Greece

**Keywords:** Bone quality and biomechanics, Motor control, Ageing, Prognostic markers

## Abstract

An ongoing thrust of research focused on human gait pertains to identifying individuals based on gait patterns. However, no existing gait database supports modeling efforts to assess gait patterns unique to individuals. Hence, we introduce the Nonlinear Analysis Core (NONAN) GaitPrint database containing whole body kinematics and foot placement during self-paced overground walking on a 200-meter looping indoor track. Noraxon Ultium Motion^TM^ inertial measurement unit (IMU) sensors sampled the motion of 35 healthy young adults (19–35 years old; 18 men and 17 women; *mean* ± 1 *s.d*. age: 24.6 ± 2.7 years; height: 1.73 ± 0.78 m; body mass: 72.44 ± 15.04 kg) over 18 4-min trials across two days. Continuous variables include acceleration, velocity, position, and the acceleration, velocity, position, orientation, and rotational velocity of each corresponding body segment, and the angle of each respective joint. The discrete variables include an exhaustive set of gait parameters derived from the spatiotemporal dynamics of foot placement. We technically validate our data using continuous relative phase, Lyapunov exponent, and Hurst exponent—nonlinear metrics quantifying different aspects of healthy human gait.

## Background & Summary

An ongoing thrust of research focused on human gait pertains to identifying individuals based on gait patterns using various analytical techniques on various data types (e.g., images, videos, and radar signals)^1–9^. The motivation behind this approach is that if the model is complicated enough (e.g., the neural network used has enough layers), then analyzing large amounts of gait data will yield the expected results. Even without any time spent *a priori* using expertise in human movement science to decide on features to include in building such model. However, these attempts are thwarted by the fact that we still do not know whether some gait features can be attributed uniquely to individuals. This knowledge is fundamental to understanding whether each individual has a unique “gaitprint” just as each individual has a unique fingerprint.

The distinctiveness of one’s gaitprint readily lends itself to personal identification. Ongoing research from our laboratory offers compelling evidence that an individual’s manner of walking is as unique and singular as a fingerprint itself^10,11^. This revelation has profound implications, spanning diverse fields. In the realm of biometrics, the utilization of gait analysis could revolutionize the identification of perpetrators at crime scenes or enable seamless access to authorized areas without disrupting the individual’s activities. Similarly, in the domain of athletics, an individual’s distinctive running style or specialized techniques geared towards achieving specific sports objectives could greatly benefit from gaitprint recognition^5^. This extremely personal identification enables the creation of tailored training regimens, honing in on the subtle nuances that differentiate athletes from their peers. However, the most transformative applications may lie within the medical arena. Personalized health monitoring has long been championed as a superior approach to rehabilitation when contrasted with symptom-based treatment plans applied broadly to patient populations. The intricacies of transient or persistent gait characteristics used for distinguishing individuals hold immense potential for crafting nuanced rehabilitation programs. These programs could offer more effective and precise interventions, warranting thorough investigation and exploration.

Stability in human performance goes hand in hand with fluctuation—even the most skilled musicians and athletes bring their lifetime of reliable practice and tune it based on the unique moment of each live performance. Perfect replication is rare and might hamper performance to meet the present circumstance or task. Variation is necessary and pervasive in well-practiced movements. In all human performance, there is a stability that thrives on and coexists with natural variability. Even for upright bipedal-walking gait, regular though our stride may be, no two footfalls are exactly the same. There is in all human performance a stability that thrives on and coexists with natural variability. Human gait variability has been specifically quantified to predict cognitive and physiological declines and prevent life-threatening consequences (e.g., identification of older adults at great risk of falling)^12,13^. For instance, variability has been studied for decades in clinical research on heart rate irregularities^14–16^, congestive heart failure, arterial blood pressure irregularities^17,18^, cerebral ischemia^19,20^, epileptic seizures^21,22^, and many other conditions^23–26^, to comprehend their complexity and eventually create prognostic and diagnostic tools. Likewise, the study of human gait variability can provide a window for understanding whether each individual has a unique “gaitprint” just as each individual has a unique fingerprint. For instance, natural fluctuations in walking (e.g., stride-to-stride fluctuations) follow a certain state of variability, which refers to the idea that human steps never exactly replicate themselves^27–29^. Therefore, we contend that the variability of human gait is the key to answering this question^30,31^. Assessing gait characteristics unique to individuals could then be used as an early indicator and predictor of disease and disability.

Movement fluctuations in healthy individuals show long-range correlations closely resembling fractional Gaussian noise (fGn) whose autoregressive coefficient *ρ* decays with lag *k* in a power-law fashion: $\rho k=\frac{1}{2}\left({\left|k+1\right|}^{2H}-2{\left|k\right|}^{2H}+{\left|k-1\right|}^{2H}\right)$^32^. The Hurst exponent, *H*, can cause the moments of the autocorrelation to diverge for $0.5 < {H}_{fGn}\le 1$; in this range, the autocorrelation function decays asymptotically towards zero, as *k* tends to infinity^32^. The fGn is often referred to as “pink noise.” Pink noise is “fractal,” or statistically self-similar with fluctuations scaling invariantly with time, as suggested by the above autocorrelation equation^33^. The lack of a pink noise structure is referred to as a “white noise” structure, or lacking long-range correlations (i.e., ${H}_{fGn}=0.5$). Finding a pink noise structure in a measurement series suggests that the underlying causal processes operate simultaneously across several timescales and provide a “persistent,” long-range correlated structure in the time-evolving behavior. So far, nonlinear analytical methods quantifying long-range correlations have been used to characterize the healthy structure of variability in different gait parameters^34–36^, and to distinguish healthy temporal structures of variability from those found during aging and disease^37–40^. These nonlinear analytical methods have never been used to identify gait characteristics unique to individuals. Any attempt to identify gait characteristics unique to individuals by leveraging variability inherent to the human gait would require a large gait dataset that, at the very least, fulfills the following conditions:

- reflects walking in everyday life (overground as opposed to walking on a treadmill)
- includes nuances necessary to provide sufficient variability in gait parameters inherent to daily walking (e.g., when taking turns, varying walking speed, etc.)
- contains huge quantities of data per individual—preferably spanning across trials to hours to days to weeks—to identify which gait parameters remain consistent within the individual and which parameters vary
- yields a variety of gait parameters—lying in the spatial, temporal, and spatiotemporal domains—to increase the chances of identifying the latent relationships among different gait parameters

However, to our knowledge, no gait dataset exists that fulfills those conditions, as evident by a sampling of existing gait datasets in Supplementary Table 1.

Supplementary Table 1 provides a survey of existing gait datasets and supports the above claim. We review only datasets published after 2010 as motion capture technology has significantly advanced in the last decade, including rapid improvements to inertial measurement unit (IMU)-based motion recording devices. Note that this survey is not exhaustive, and we may have missed several important gait datasets, but the sole purpose of this exercise is to put the present gait dataset in context and to provide interested readers with a glimpse of state-of-the-art datasets. These datasets provide critical gait data in multitudinous walking conditions from multiple countries spanning all around the globe and are fueling gait research in meaningful ways, as affirmed by the thousands of citations they have accumulated. However, these datasets also have several limitations that preclude their utility for assessing gait characteristics unique to individuals using the most advanced nonlinear analytical methods:

- **A singular focus on straight walking**. With a few exceptions^41^, almost all existing gait datasets focus on straight walking over an even surface. Straight walking is not fully representative of real-life walking, which involves curved paths, sharp turns, uneven surfaces, and countless obstacles. During curved walking, for example, a combination of motor strategies come into play to stabilize the head in space and orient the entire body in the desired direction^42,43^. Consequently, curved walking challenges spatiotemporal, stability, and symmetry-related gait patterns^44–46^. A gait dataset representative of real-world walking must have at least a few elements of curved walking.
- **Lack of dynamic optic flow**. Existing gait datasets mostly constitute data collected while walking on the treadmill in a gait laboratory. As opposed to walking in a natural environment where optic flow is highly dynamic, optic flow while walking on a treadmill is relatively static. Treadmill walking has additional constraints such as limited space to walk, possible dangers if the subject is too far back or too close to the side, and a velocity vector from the belt that is continuously added to the subject’s velocity vector. Given the critical role of optic flow in the perceptuomotor control of walking^47,48^ and the role of these constraints on moderating walking dynamics^49–54^, these datasets miss a very important component influencing gait patterns. A more useful gait dataset must be based on walking in a dynamically changing visual environment.
- **Reliance on laboratory-grade movement registration devices**. Many existing gait datasets have been collected using expensive, laboratory-grade movement registration devices (e.g., instrumented treadmills, and optical motion trackers with multiple cameras). Often, this gold standard gait research may cost upwards of a hundred thousand dollars. There is no doubt that these devices provide high-precision and high-accuracy recordings with potentially high impact. However, not all institutions and investigators can receive consistent and substantial funding to be able to have access to these facilities. At the same time, a reliance on those laboratory-grade devices affects practitioners whose subjects may not be able to enter the laboratory due to injury, disease, or disability. These factors severely limit the expansion and utility of these datasets. To circumvent these challenges, a gait dataset, collected using a portable and more easily accessible yet accurate motion registration device, allows systematic expansion of the previous datasets to subjects restricted to in home or assistive-care settings and comparison with future datasets.
- **Emphasizing sample size over an individual**. The typical approach to existing gait datasets has been to focus on breadth (e.g., a large number of subjects) at the cost of depth (e.g., a large amount of data per subject). The motivation behind this approach is a habit of blindly analyzing a wide array of shallow data without using human movement expertise to decide on included features to build appropriate models a priori. Nonetheless, a gait dataset appropriate for assessing gait characteristics unique to individuals must also involve a large amount of data per individual and domain expertise (e.g., biomechanics, human movement variability) to train models with carefully curated features of gait that have proven track records of characterizing human movements.
- **Limited scope for analyses**. A major drawback of the existing gait datasets is the small number of gait parameters used or calculated. For instance, joint angle trajectories are not enough to determine step width variability, which are significant predictors of fall risk in older adults^55–63^, and can differentiate older adult fallers from non-fallers after a slipping event^64^. Likewise, a dataset singularly focused on foot placement variables (e.g., step length, stride length, step width) is not fully equipped to investigate the coordination patterns underlying specific features of imperceptible gait pattern fluctuations. On the other hand, a comprehensive study of gait variability can incorporate as many as 16 gait parameters at the scale of individual steps^65^ in addition to the joint angle amplitudes, velocities, and accelerations belying these gait parameters. The literature is hungry for a gait dataset that collates all these kinematic variables and gait parameters for the same set of individuals to produce a well-rounded investigation of unique gait characteristics—an individual’s “gaitprint.”
- **Test-retest reliability**. Test-retest reliability is a measure of reliability obtained by administering the same test twice over a period of time. The *mean* and *standard deviation* may not vary, but the temporal structure may vary across trials. Some previous studies have assessed the retest reliability of gait parameters and found them to be high for many investigated parameters^66–71^. Notwithstanding, existing gait datasets fail to repeatedly sample subjects across a sufficient timeframe (e.g., days and weeks) to assess test-retest reliability. Furthermore, these datasets cannot identify which gait patterns are unique to the individual and how robust gait patterns are after a retest has been completed. A gait dataset with many trials for each subject collected over two sessions with a rest period of multiple days is necessary to distinguish between reliable and unreliable gait parameters.

The present dataset—which we call the Nonlinear Analysis Core (NONAN) GaitPrint dataset—overcomes all the above-mentioned limitations, providing a valuable resource for assessing gait characteristics unique to individuals based on established principles of human movement variability^27–29^. The database provides whole-body kinematics and foot placement variables during self-paced overground walking on a 200-meter looping indoor track. Noraxon Ultium Motion^TM^ IMUs sampled the motion of 35 healthy young adults (19–35 years old) at 200 Hz over 18 4-min trials across two days. Many discrete and continuous variables such as velocity, acceleration, and cadence are outlined in the section “Data processing and extraction of foot placement variables.”

## Methods

### Subjects and ethical requirements

Thirty-five adults (18 men and 17 women; *mean* ± 1 *s.d*. age: 24.6 ± 2.7 years; height: 1.73 ± 0.78 m; body mass: 72.44 ± 15.04 kg) participated in the present study. Subjects were identified and recruited by word of mouth, campus-wide emails, and Facebook/Twitter posts that link to webpages owned by the University of Nebraska at Omaha. This study was also allowed to use recruiting flyers distributed throughout the community at universities, clinics, health centers, gyms, fitness classes, libraries, cafes/coffee shops, retirement homes, stores, and community bulletin boards. Subjects were paid $20 per session for participation. Interested individuals were recruited only if they (i) were able to provide informed consent; (ii) were able to walk independently without an assistive device; (iii) did not self-report diagnosis of neurological disease; and (iv) did not self-report diagnosis of any lower limb disability, injury, or disease. Subjects provided verbal and written informed consent approved by the University of Nebraska Medical Center’s Institutional Review Board (# 0762-21-EP). This study does not contain sensitive data (i.e., from children) and all participants were between 19 to 31 years old^72–85^.

### Sample size justification

We expect that many of the statistical analyses performed on the present dataset will involve either statistical comparisons distinguishing between clinical groups (e.g., young vs. older adults, healthy adults vs. stroke survivors) or machine learning to identify distinguishing gait patterns among individuals. Excitingly, this dataset constitutes the first batch of subjects from an ongoing project that involves five other populations: healthy middle-aged adults (36–55 years old); healthy older-aged adults (56 + years old); lower-limb amputees; post-stroke patients; and patients with peripheral arterial disease (PAD). Hence, the issue of statistical power can be addressed by first considering the magnitude of differences between any of these groups that can be detected with reasonable probability. To address this issue within the context of mixed-effects models—which constitute the most common method for linear modeling, we simulated datasets (*n* = 1,000) based on effect sizes reported in a recent meta-analysis that identified systematic differences in young and old adults in terms 1/*f* characteristics^86^. The same study also compared Parkinson’s patients with healthy controls. We used typical values and effect sizes from this study to simulate the multilevel data structure implied in the design of the present study. We conservatively focused the simulations on comparing young and old adults by reasoning that the effect sizes due to age were smaller than those due to clinical group^86^. Based on these simulations, a sample size of 30 healthy adults will provide 98% power to detect a standardized effect size of 0.20 (a small effect defined by Cohen^87^), assuming a 5% type 1 error rate. Hence, the current sample size of 35 healthy adults should allow reliable comparison with previously published datasets. Finally, to fully characterize each individual’s gait patterns, instead of collecting a limited amount of data for a larger number of subjects, we decided to collect a huge amount of data per subject over multiple days for a modest number of subjects to confer benefits to future analyses.

### Setup and procedures

Kinematic data were collected during self-paced overground walking on a 200 m indoor track at the University of Nebraska at Omaha on two separate days spread one week apart (one subject—identified as S017—did not return for the second session). Each session lasted for a maximum of two hours and thirty minutes. Subjects were requested to wear semi-tight athletic clothing and shoes that they were comfortable walking in for no longer than 2.5 hours. Subjects were screened and consented at the Center for Research in Human Movement Variability (MOVCENTR), housed within the Biomechanics Research Building at the University of Nebraska at Omaha. Per session, subjects performed nine four-minute overground walking trials at their self-selected walking speed on an indoor running/walking track at the adjacent Health & Kinesiology Building. The trials were spread over three blocks of three trials each, with a self-chosen resting period between consecutive blocks of up to five minutes.

**Fig. 1:**
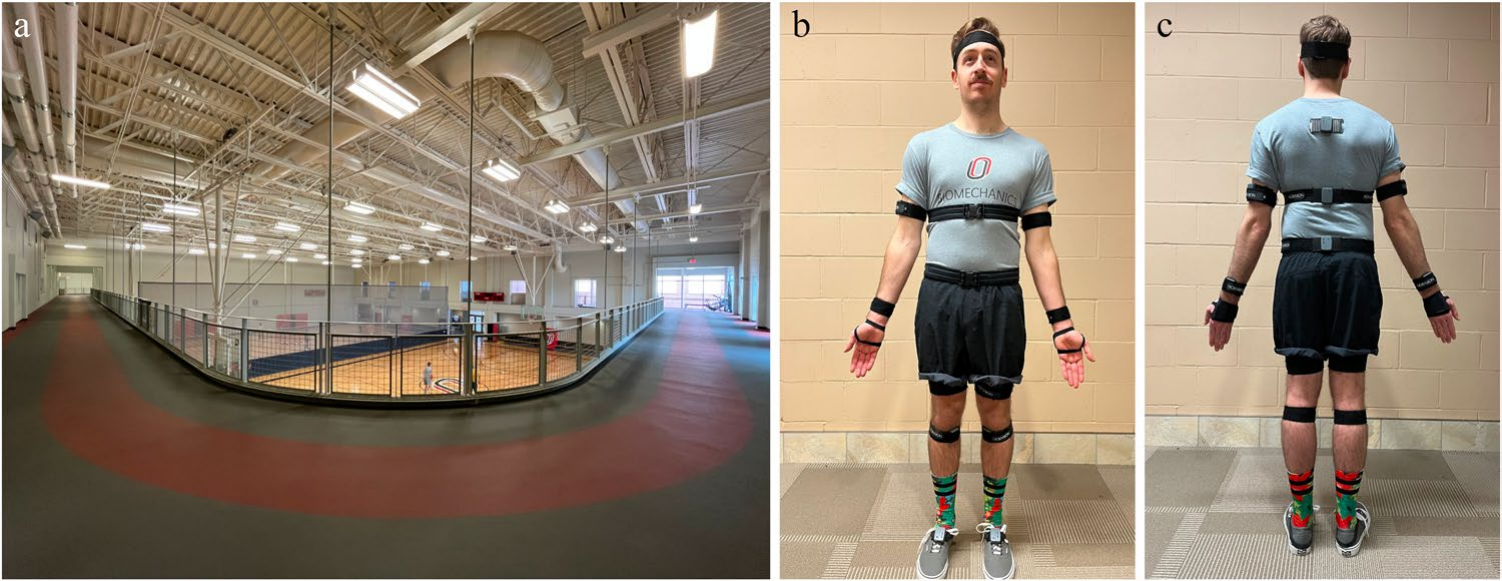
Experimental setup. (**a**) Indoor running/walking track where the data was collected. (**b,c**) Anterior and posterior views of a model wearing the Noraxon Ultium MotionTM IMU sensors prior to walking on the track shown in (**a**) The model in (**b,c**) is the first author, not a participant, and approves the use of his image.

Kinematic data during walking were collected at 200 Hz using Noraxon Ultium Motion^TM^ IMUs (Noraxon, Inc., Scottsdale, AZ). Sensors were placed on the following parts of the subject’s body, as recommended by Noraxon: head (middle of the back of the head), upper thoracic (below C7 in line with the spinal column, but high enough to not be affected by upper trapezius muscle movement), lower thoracic (in line with the spinal column at L1/T12; strap belt was positioned on lower ribs on the front side of the body), pelvis (body area of the sacrum), upper arm (midway between the shoulder and elbow joints, lateral to the bone axis), forearm (posterior and distal, where there is a low amount of muscle tissue), hand (dorsal), thigh (frontal and distal half, where there was a lower amount of muscle displacement during motion), shank (front and slightly medial to be placed along the tibia medial surface of the tibias), and foot (upper foot, slightly below the ankle; Fig. 1). All sensors, except those attached to the foot, upper thoracic, lower thoracic, and pelvic, were clipped onto a Velcro strap tightened around the respective body segment to ensure minimal motion artifact but loose enough to promote movement. At the foot, the sensor was clipped into a rubber strap going over the dorsal side of the foot wrapped around the back of the heel and under the arch of the foot. The pelvis and thoracic sensors were clipped into a small plastic clip before placement. The upper thoracic sensor was taped to the upper thoracic area on subjects’ clothing or, when possible, on the subject’s skin. At the lower thoracic and pelvic area, the sensor and platform were secured to a clipped strap tightened around the torso.

Subjects’ body mass, height, and the following anthropometric measures were collected before the first experimental session to reconstruct and scale the biomechanical gait model in the MyoResearch 3.18.126 software: skull height (mental protuberance to skull vertex), shoulder width (inter-acromion joint distance), lumbar + thoracic (C7 to S1), pelvis width (inter-anterior superior iliac spine distance), upper arm (acromion process to lateral humeral epicondyle), forearm length (lateral humeral epicondyle to radial styloid process), hand length (ulnar notch to the tip of the third phalange), thigh length (greater trochanter to lateral femoral epicondyle), shank length (lateral femoral epicondyle to lateral malleolus), and foot length (length of show). Several validation studies have shown that the kinematic data obtained from the Noraxon IMU sensors closely match the kinematic data obtained using traditional infrared motion capture systems^88–92^.

A functional walking calibration procedure was completed before each trial. First, subjects stood still for 2.5 seconds with their arms at their side and their feet close to shoulder-width apart. They then walked for 15 seconds at a self-selected pace, made a 180° turn, walked back to the starting position, and stood still for another 2.5 seconds in the same initial posture. Next, subjects were instructed to walk for four minutes at a self-selected pace that they could maintain for at least 30 minutes and to maintain that same speed throughout the four-minute trial. Subjects walked on the track either clockwise or counterclockwise, with the order of the direction decided by the rules of the track (e.g., trials completed on Monday, Wednesday, Friday, or Sunday were walked clockwise; in 423 trials (69.12%) the participants walked on the track clockwise and in the 189 trials (30.88%) they walked counterclockwise). Two investigators always walked behind subjects with a cart holding the Ultium Motion receiver connected to a laptop computer while simultaneously monitoring data quality and subject safety. At the end of each four-minute trial, subjects were verbally instructed to stop walking. They then returned to the initial position for calibration, following which the subsequent trial began. All subjects started to walk from the same position on the track. A five minute rest period was given every three trials if needed.

### Data processing and extraction of foot placement variables

Before exporting the data for the present database, the following post-processing options were applied by the Noraxon MyoResearch 3.18.126 software. First, the processing fusion mode was set to “standard”—the default fusion setting that uses an adaptive filtering technique to optimize sensor tracking while considering the cleanliness of the magnetometer data while recording. Course Stabilization was set to “Foot, Shank, Thigh, Spine,” which acts as a high-pass filtering operation on the secondary joint angles to remove sensor drift using a ten-second sliding window. This applied stabilization is recommended for severe magnetic interference affecting the whole body and stabilizes the foot, shank, and thigh from the top down. It also stabilizes the upper and lower spine segments relative to the pelvis. The final setting, progression, was set to “translation.” Secondary knee angles were turned on to determine the left and right knee abduction and external knee rotation. Acceleration was set to “sensor-based” to capture acceleration data with respect to the coordinate frame of each sensor. The software detected heel contacts using the gyroscope and accelerometer data of the foot sensors to determine stance and swing as an on/off signal. The Noraxon MyoResearch 3.18.126 software also provides an anti-wobbling correction that was applied to smooth the data at 5 Hz with a 300 ms residual to remove soft tissue artifacts.

For each trial, the following variables were exported by the Noraxon MyoResearch 3.18.126 software: the acceleration, velocity, position, and orientation of each sensor and the acceleration, velocity, position, orientation, and rotational velocity of each corresponding body segment, and the angle of each respective joint.

For each trial, the following gait parameters, units in parentheses, were determined using the filtered time series data, as defined previously^31,65^:

*Spatial parameters*

- Step length (cm)—the distance from one heel strike to the next heel strike of the opposite foot.
- Stride length (cm)—the distance between two consecutive heel strikes of the same foot.
- Step width (cm)—the lateral distance between the heel center of one heel strike and the line joining the heel center of two consecutivie heel strikes of the opposite foot.
- Distance traveled (m)—the distance the subject traveled as tracked by the pelvis sensor.

*Temporal parameters*

- Cadence (steps/min)—the number of steps per minute, also referred to as step rate.
- Step time (s)—the time elapsed from the initial contact of one foot to the initial contact of the opposite foot.
- Stride time (s)—the time elapsed between the initial contacts of two consecutive footfalls of the same foot.
- Stance time (s)—the time elapsed between the first and last contacts of a single footfall (the stance phase starts at heel contact and ends at toe off of the same foot).
- Swing time (s)—the time elapsed between the last contact of the current footfall and the first contact of the following footfall of the same foot (the swing phase starts with toe off and ends with the first contact of the same foot).
- Single support time (s)—the total time one foot is in contact with the ground throughout the gait cycle.
- Double support time (s)—the total time both feet are simultaneously in contact with the ground throughout the gait cycle.

*Temporophasic parameters*

- Stance time (%Stride time)—stance time normalized to stride time.
- Swing time (%Stride time)—swing time normalized to stride time.
- Single support time (%Stride time)—single support time normalized to stride time.
- Double support time (%Stride time)—double support time normalized to stride time.

*Spatiotemporal parameters*

- Gait speed (m/s)—the ratio of distance walked and trial time.
- Stride speed (m/s)—the ratio of stride length and stride time.

The MATLAB (Mathworks, Inc., Natick, MA) script used to determine these gait parameters—GaitPrint_Spatiotemporal_Calculation.m—is provided as part of the database on figshare.

## Data Records

All data is made available using Figshare^93^. The deidentified subject information is stored in the file named GaitPrint_Subject_Characteristics.csv, describing for all subjects the screening outcomes, age, sex, body mass, height, hand and food dominance, shoe make and model, and anthropometrics measures: skull height, shoulder width, lumbar + thoracic, pelvis width, upper arm, forearm length, hand length, thigh length, shank length, and foot length. An additional file, GaitPrint_Trial_Characteristics.csv, described for all subjects the session date and start time, shoe make and model, walking direction, start time, first step taken, and any notes taken during the trial. All kinematic data have been grouped into subject-specific folders and .csv files, each file bearing the subject’s alphanumeric code (e.g., S001_G01_D01_B01_T01). All data files have a tabular structure with the following headers:

### ID

This field codes the subject number. It is labeled “S001,” “S002,” “S003,” … for the 35 subjects.

### Group

This field codes the study population. It is labeled “G01” for the current dataset.

### Day

This field codes whether the data was collected during the first or second day. It is labeled “D01” and “D02” for Day 1 and Day 2, respectively.

### Block

This field codes the block number within a session. It is labeled as “B01,” “B02,” and “B03” for Block 1, Block 2, and Block 3, respectively.

### Trial

This field codes the trial number within a block. It is labeled as “T01,” “T02,” and “T03” for Trial 1, Trial 2, and Trial 3, respectively.

### S###.zip (Raw Data)

These folders contain one .csv file for each trial completed by the participant. For example, S001.zip contains 18 .csv files each as a table providing all the raw data from that subject’s trial. The data are arranged in a matrix of 48,000 rows by 321 columns, with one row per timestamp. The first column, “Time” provides the timestamps, in milliseconds. The next 320 columns each provide the kinematic variables exported by the Noraxon MyoResearch 3.18.126 software: the acceleration, velocity, position, and orientation of each sensor, and the acceleration, velocity, position, orientation, and rotational velocity of each corresponding body segment, and the angle of each respective joint.

### Spatiotemporal_variables.zip

This folder contains one .csv per trial where each file contains a table providing all the spatiotemporal variables listed above. The data are arranged in 26 columns, with one row per sample in the time series of each gait parameter. cadence (steps/min), step time (s), left step length (cm), right step length (cm), left step width (cm), right step width (cm), left stride length (cm), right stride length (cm), left stride time (s), right stride time (s), left stance time (s), right stance time (s), left swing time (s), right swing time (s), single support time (s), double support time (s), left pct stance (%GC), right pct stance (%GC), left pct swing (%GC), right pct swing (%GC), pct single (%GC), pct double (%GC), average speed (m/s), left stride speed (m/s), right stride speed (m/s), and distance traveled (m).

We acknowledge that numerous researchers, particularly those affiliated with teaching institutions, may encounter obstacles when delving into the analyses of spatiotemporal variables or raw data. These hurdles may include limited access to paid software tools or a potential deficiency in coding, particularly in languages such as Python or R. To foster accessibility and facilitate secondary research endeavors involving this data, we have taken the initiative to offer all spatiotemporal variables and raw data for each subject and trial as distinct .csv files. Spatiotemporal files are conveniently bundled within a compressed folder titled “Spatiotemporal_Variables.zip” and each subject’s raw data within compressed folders titled “S###.zip.” In addition, we have provided a compressed folder titled “template_scripts.zip” containing basic code examples with MATLAB, Python, and R extensions. This approach serves to lower the barrier to entry, enabling a wider range of researchers to engage with the data effectively.

## Technical Validation

### Continuous phase relationship between the right and left limb segments reveals highly consistent inter-limb coordination over multiple gait cycles

The lower extremity segments of a walking individual can be considered a coupled system, and the interaction between the segments effectively moves the body forward. The behavior of such a dynamical system can be described by plotting a variable versus its first derivative—the phase portrait—that quantifies human movement^94^. The phase portraits of limb segments resemble a limit cycle system because the coordination is cyclic and dissipative, requiring energy to maintain the behavior^95^. Accordingly, the relation between two limb segments in phase space, or relative phase, describes the dynamic coordination of these variables^96–98^. Continuous relative phase, *Φ*, describes the phase space relation between two segments in a form of a low-dimensional variable that reflects changes in phase relationship throughout the movement. Hence, this makes continuous relative phase an appealing variable to assess inter- and intra-limb coordination^99^. *Φ* was defined for the following joint pairs: (i) right and left thigh pitch angle, (ii) right and left shank pitch angle, and (iii) right and left foot pitch angle, for each gait cycle^100^. Angular position data were first normalized to 100 data points, and centered so that the calculated segment phase angles are oriented around 0° (${x}_{centered}=x\left({t}_{i}\right)-min\left(x\left(t\right)\right)-\left(max\left(x\left(t\right)\right)-min\left(x\left(t\right)\right)\right){\rm{/}}2$). Then, to minimize the error caused by the phase portrait method, analytical signals were obtained via Hilbert transform ($\zeta \left(t\right)={x}_{centered}\left(t\right)+iH\left(t\right)$). The segment phase angle of each data point was calculated using the real part and the imaginary part that are computed through the Hilbert transform (${\varphi }_{segment}\left({t}_{i}\right)=arctan\left(H\left({t}_{i}\right){\rm{/}}x\left({t}_{i}\right)\right)$). *Φ* was calculated as the difference between the right and left segment phase angles at each time point ${\Phi }({t}_{i})={\varphi }_{rightsegment}({t}_{i})-{\varphi }_{leftsegment}({t}_{i})$. When *Φ* is 0°, it indicates that the phase space relationship between the segments is symmetrical and is called in-phase. On the other hand, when *Φ* is 180°, the two segments are in an anti-phase relation, i.e., out of phase. *Φ* can capture changes in limb coordination across the gait cycle of healthy adults^101,102^, the effects on inter- and intra-limb limb coordination due to cognitive load and walking speed^103,104^, changes associated with development^105^ and aging^106^, neuropathy^107^, injury^108^, recovery post-injury^109,110^, and amputation^103^. ${\Phi }$ was calculated across all gait cycles within each self-paced overground walking trial.

**Fig. 2:**
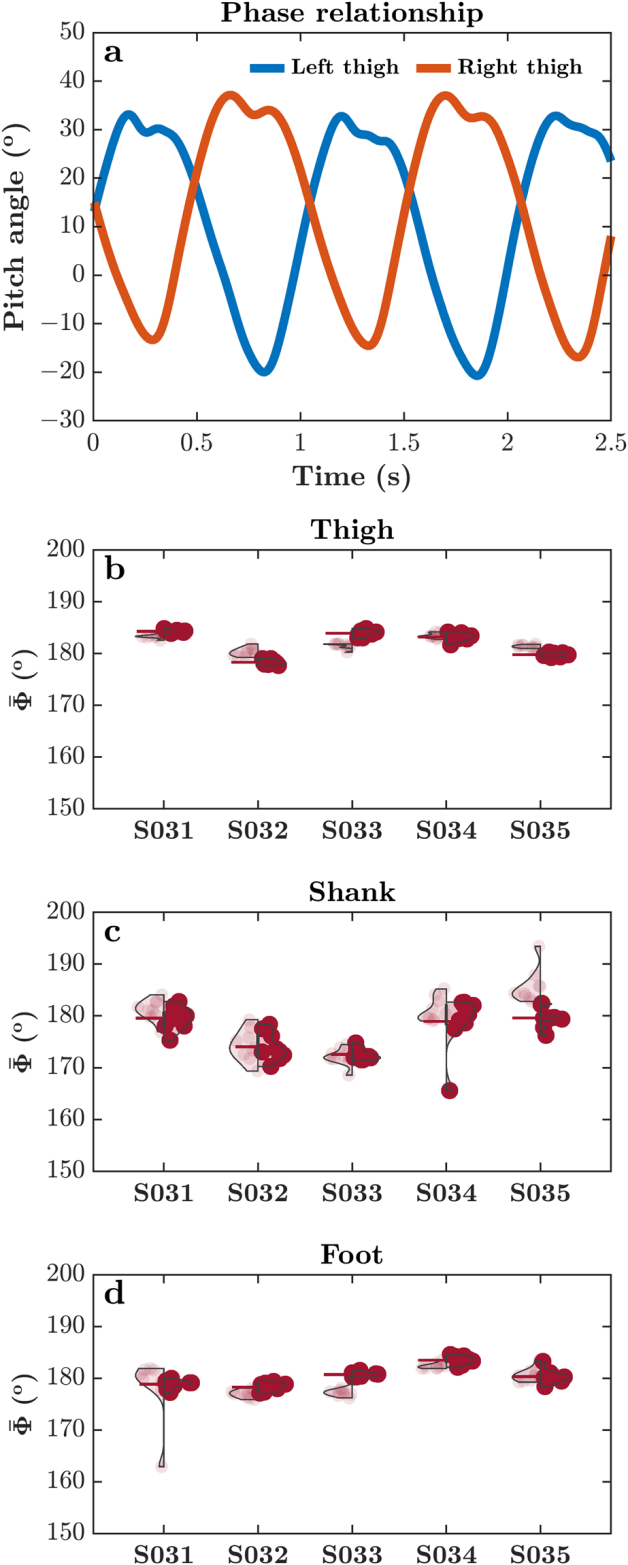
The right and left segment pitch angles showed an out-of-phase continuous phase relationship. (**a**) A representative sample of the right and left thigh pitch angle used to calculate the phase relationship between the right and left limbs. (**b**–**d**) Violin plots of the continuous relative phase, $\bar{{\Phi }}$, across all gait cycles within a trial between the right and left segment pitch angles for five representative subjects. (**b**) Violin plots of $\bar{{\Phi }}$ for thigh pitch angle. **c**. Violin plots of $\bar{{\Phi }}$ for shank pitch angle. (**d**) Violin plots of $\bar{{\Phi }}$ for foot pitch angle. Each violin plot shows $\bar{{\Phi }}$ for the two days (Day 1 on left, and Day 2 on right)_._ As expected, $\bar{{\Phi }}$ for all three segment pairs hover around 180°, indicating an out-of-phase relationship between the right and left limb segments. Additionally, the high intra-individual consistency in $\bar{{\Phi }}$ between the two days further strengthens our assertion about the validity of our data. Vertical lines represent the interquartile range of relative phase, and colored horizontal bars represent the *mean* of $\bar{{\Phi }}$ (*n* = 9 trials) for the respective subject/day.

Figure 2b–d show violin plots of $\bar{{\Phi }}$ between the right and left segment pitch angles for thigh, shank, and foot for five representative subjects. The obtained $\bar{{\Phi }}$ for all three segment pairs hover around 180°, indicating an expected out-of-phase relationship between the right and left limb segments characteristic of healthy gait. Additionally, the high intra-individual consistency in $\bar{{\Phi }}$ between the two days further strengthens our assertion about the reliability of our data. This session-to-session reliability of the obtained $\bar{{\Phi }}$ point toward the uniqueness of individual gait patterns across both sessions^111^.

**Fig. 3:**
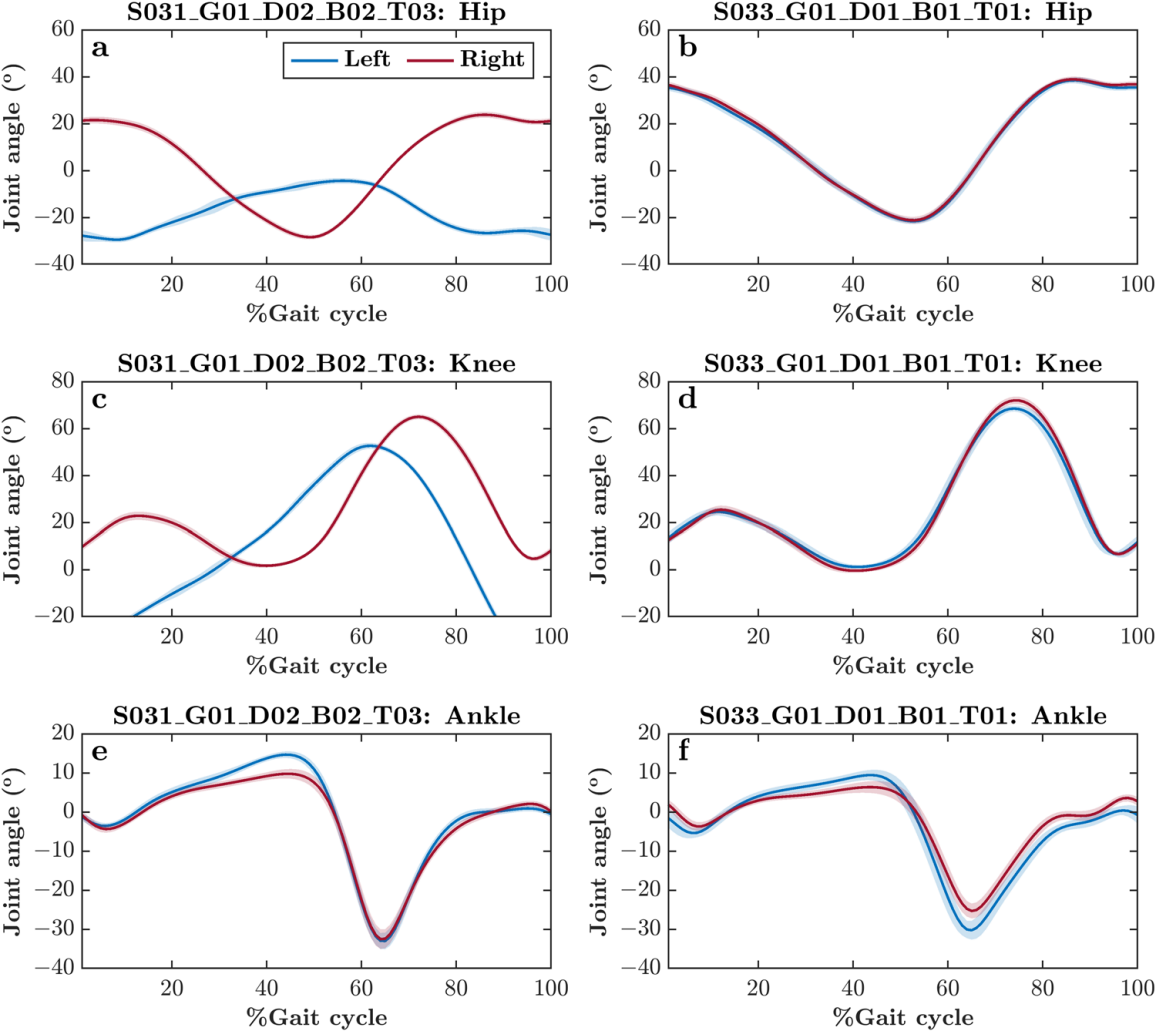
*Mean* joint angle trajectories across all gait cycles within a self-paced overground walking trial for two representative subjects, one with anomalous $\bar{{\Phi }}$ between the right and left ankle pitch angles (Subject 031; (**a,c,e**)) and the other with typical $\bar{{\Phi }}$ between the right and left ankle pitch angles, as shown in Fig. 4d (Subject 033; (**b,d,f**)), as shown in Fig. 2. Shaded areas indicate *standard deviation* across all gait cycles. Note the altered *mean* joint angle trajectories and greater *standard deviation* across all gait cycles in the subject with anomalous $\bar{{\Phi }}$ between the right and left ankle angles (i.e., left vs. right panels).

While most subjects showed $\bar{{\Phi }}$ close to 180°, we found a total of 13 trials across four subjects with $\bar{{\Phi }}$ deviating considerably (e.g., Subject 031, as indicated in Fig. 2d). To understand the cause of these anomalous $\bar{{\Phi }}$, we explored these trials in further detail. We found altered *mean* joint angle trajectories across all gait cycles within these trials, as well as a greater *standard deviation* across all gait cycles (see joint angle trajectories from one such anomalous trial—for Subject 031—contrasted with a valid trial; Fig. 3). Hence, the continuous phase relationship between the right and left limb segments was also diagnostic of data quality.

### Lyapunov exponent (*λ*_1_) values reveal highly consistent trajectories over multiple gait cycles

**Fig. 4:**
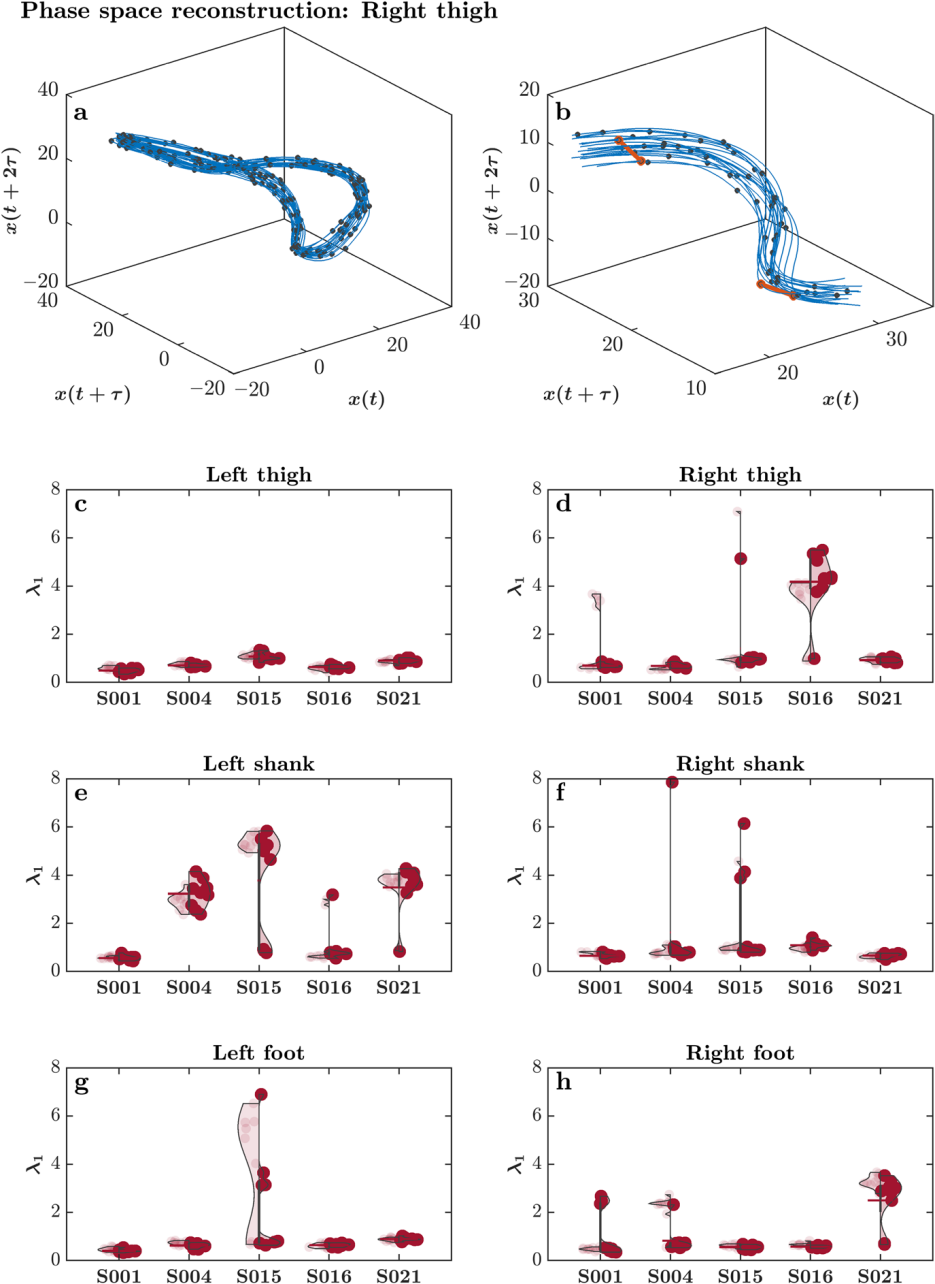
The largest Lyapunov Exponent, *λ*_1_, describes the limb segment’s diverging trajectories over multiple gait cycles. The motion of thigh, shank, or foot over multiple strides can be depicted as a series of neighboring trajectories in a time-delay embedding. The embedding dimension is calculated from the False Nearest Neighbors algorithm, which detail the number of time delay copies required to accurately replicate movement trajectory dynamics (i.e., an attractor). This example shows an embedding dimension of 3 (original plus 2 copies), for viewing purposes. The time, *τ*, is determined through the Average Mutual Information algorithm. (**a**) Each of the time series (original plus copies) are plotted to provide the reconstructed state space attractor. This is an example of the thigh segment angle time series embedded into 3 dimensions. (**b**) Inset from a showing the calculation of the Lyapunov exponent. Conceptually, a single point along a trajectory and its true nearest neighbor are selected, shown by a red line connecting two dots in the upper left corner of (**b**) and the Euclidean distance between these points is calculated (d*t*). These points are then followed along their respective trajectories for a certain number of time points (*n*), followed by distance recalculation (d*t*’, shown by red lines connecting red dots). A new true nearest neighbor is selected, shown by a red line connecting two dots in the bottom right corner of (**b**) and the process is repeated. The log of the ratio of these distances is calculated and then normalized to the time the points traversed through the trajectory to yield the Lyapunov exponent. A smaller *λ*_1_ indicates higher predictability, or less divergence, of the dynamical system. (**c**–**h**) Violin plots of *λ*_1_ for the segment pitch angles for five representative subjects. (**c**) Left thigh. (**d**) Right thigh. (**e**) Left shank. (**f**) Right shank. (**g**) Left foot. (**h**) Right foot. Each violin plot shows *λ*_1_ for the two days (Day 1 on left, and Day 2 on right). As expected, these *λ*_1_ indicate gait cycle sensitivity that is dependent on initial conditions and is characteristic of healthy walking. Additionally, the high inter-individual variation in *λ*_1_ is accompanied by high intra-individual consistency in *λ*_1_ between the two days, further strengthening our assertion about the validity of our data. Vertical lines represent the interquartile range of *λ*_1_, and colored horizontal bars represent the *mean* of *λ*_1_ (*n* = 9 trials) for the respective subject/session.

Walking, in part, results from the repetitive rotation of limb segments around a joint (e.g., ankle, knee, hip) which is a cyclical process. This repetition creates trajectories in three-dimensional space that can be tracked, recorded, and plotted by motion capture equipment and software. One way to study the variability of walking trajectories is to inspect how the repetitive rotation of these joints changes over many gait cycles^27^. That is, do limb segments trace out the exact same trajectory over multiple gait cycles? Or, do movement trajectories deviate over time? That is, do movements converge to a common trajectory, or do they diverge? The (Largest) Lyapunov Exponent (*λ*_1_) provides a direct measure of the divergence of trajectories by examining trajectory behavior within the so-called reconstructed “state space attractor”—a set of states that a dynamical system tends to evolve toward over various initial conditions^112–115^ (Fig. 4a,b). As such, it is one aspect of measuring the stability of system’s time-evolving dynamics. Technically, *λ*_1_ measures the rate at which trajectories diverge over gait cycles. This rate reflects the unique functional organization of each person’s neuromuscular system^116^. Changes in this tendency to converge or diverge should reflect how a walker adapts to perturbations during locomotion and hence, qualitative changes in movement trajectory dynamics could reveal the onset of pathology^117–121^. We used Wolf *et al*.’s algorithm^112^ to assess *λ*_1_.

Figure 4c–h show violin plots of *λ*_1_ for segment pitch angles at the thigh, shank, and foot for five representative subjects. The obtained *λ*_1_ is rather small and within close approximation to the values reported in several previous studies^122^, indicating highly predictable gait cycles characteristic of healthy walking. We would like to also note that while some previous studies reported even smaller *λ*_1_^123–125^, these studies used joint angles for estimating *λ*_1_ as opposed to segment pitch angles used in the present dataset. Additionally, we observe high inter-individual variation in *λ*_1_, accompanied by high intra-individual consistency in *λ*_1_ between the two sessions. This session-to-session test-retest reliability of the obtained *λ*_1_ points toward the uniqueness of individual gait patterns across both sessions^111^.

**Fig. 5:**
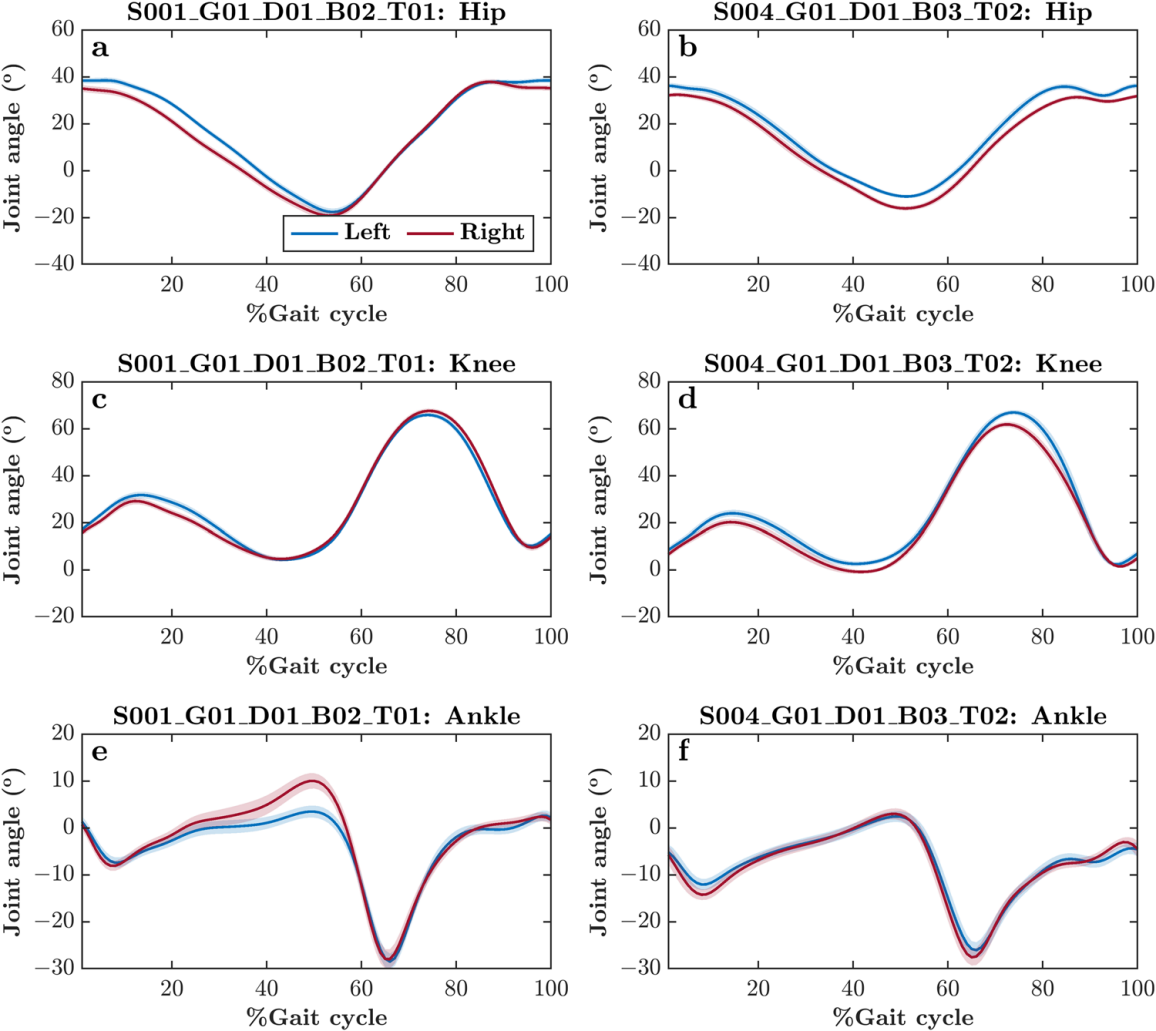
*Mean* joint angle trajectories across all gait cycles within two representative self-paced overground walking trials. Both subjects show *λ*_1_ close and far, from the *mean* as shown in Fig. 4. Shaded areas indicate *standard deviation* across all gait cycles. Note that despite significant anomaly in *λ*_1_, the *mean* joint angle trajectories and *standard deviation* across all gait cycles can be atypical or irregular across trials (i.e., left vs. right panels).

Several subjects showed large variations in *λ*_1_ across trials (e.g., *λ*_1_ for the right thigh for Subject 001; Fig. 4d). To understand whether this variation reflects anomalous levels of divergence across trajectories, we explored such trials in further detail. We found relatively normal *mean* joint angle trajectories across all gait cycles within these trials (see joint angle trajectories from one such pair of trials; Fig. 5). Hence, we posit that these highly variable *λ*_1_ reflect overground walking on a looping track, which imposes fewer constraints on gait than a treadmill, and evokes greater divergence among linked segment angles in high-dimensional space due to a wider walking space and the presence of turns. This heightened sensitivity of *λ*_1_ to individuals’ responses to gait constraints also means that *λ*_1_ could serve as a more reliable metric—or an important member of a set of metrics—for assessing gait patterns unique to individuals.

### Close-to-one hurst exponent, *H*_*fGn*_, values indicate the pink noise structure in the time series of stride length and stride time

**Fig. 6:**
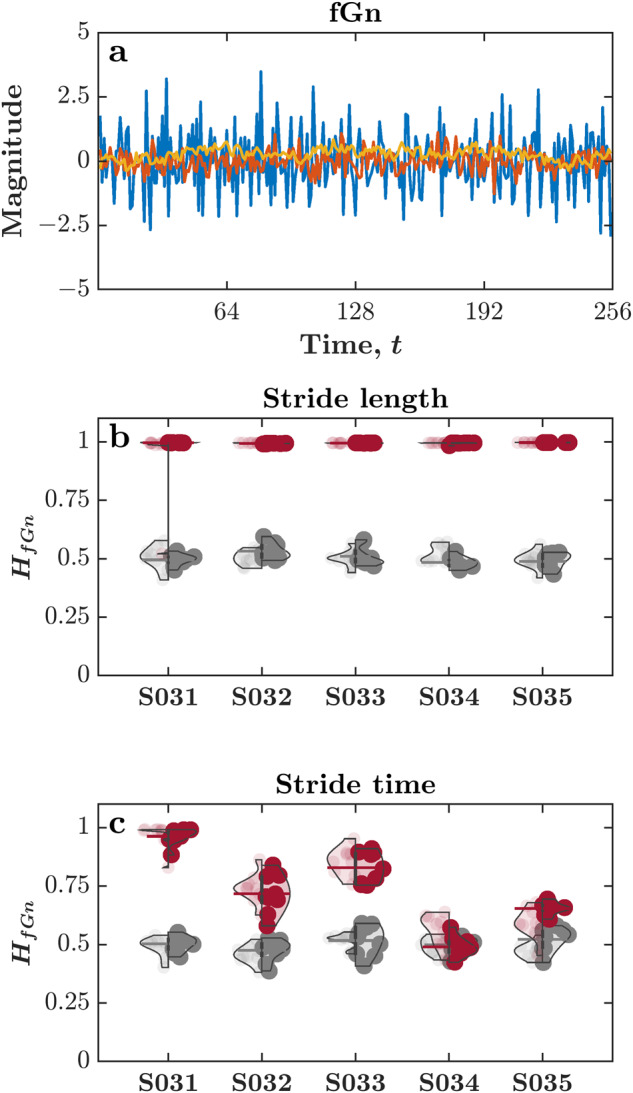
Hurst exponent, *H*_*fGn*_, values indicate the pink noise structure in stride length and stride time characteristic of a healthy gait. (**a**) The fractional Gaussian noise (fGn) time series for *H*_*fGn*_ = 0.1, 0.5, and 0.9 in blue, red, and yellow, respectively. (**b**) Violin plots of *H*_*fGn*_ values calculated using stride length for five representative subjects for the original (red) and shuffled (grey) time series. **c**. Violin plots of *H*_*fGn*_ values for stride time for five representative subjects for the original (red) and shuffled (grey) time series. Each violin plot shows *H*_*fGn*_ values for the two days (Day 1 on left, and Day 2 on right). As expected, *H*_*fGn*_ values for both the time series of Stride length and Stride time resemble pink noise structure and shuffling the original series yields *H*_*fGn*_ values resemble white noise. Additionally, the high inter-individual variation in *H*_*fGn*_ values is accompanied by high intra-individual consistency in *H*_*fGn*_ values between the two sessions, further strengthening our assertion about the validity of our data. Vertical lines represent the interquartile range of *H*_*fGn*_ values, and colored horizontal bars represent the *mean* of *H*_*fGn*_ values (*n* = 9 trials) for the respective subject/session.

The optimal movement variability hypothesis suggests that human gait variability (i.e., the fact that human steps never repeat themselves exactly) exhibits certain characteristics and optimal forms typically found in healthy individuals^27,28,126^. Measurements of physiological, motor, and cognitive variability in healthy individuals show varying degrees of long-range correlations^127–131^, and their loss due to aging and disease^23–26^. The Hurst exponent *H* captures long-range correlations in terms of the moments of the autocorrelation, which diverge for $0.5 < {H}_{fGn}\le 1$^32,132^ (Fig. 6a). An fGn time series with *H*_*fGn*_ < 0.5 is anti-persistent, i.e., an increase will most likely be followed by a decrease and vice-versa. This means that future values tend to return to a long-term *mean*. An fGn time series with *H*_*fGn*_ = 0.5 resembles a random walk, implying no correlation between the current and future values. An fGn time series with *H*_*fGn*_ > 0.5 is persistent, i.e., an increase will most likely be followed by another increase and vice-versa. The very notion of the *mean* is meaningless for persistent time series. As mentioned above, biologists and psychologists have encountered the mathematical structure of *H*_*fGn*_ > 0.5 under the label of “1/*f* noise” or “pink noise” (e.g.^133,134^). *H*_*fGn*_ or alternatively, the fractal scaling exponent *α*, has been used extensively to analyze the temporal structure of gait variability during walking and running, while locomoting overground or on a treadmill^135–137^. The values of *H*_*fGn*_ depend on task constraints such as the walking speed^138,139^ and can distinguish between healthy gait in young adults and altered gait due to aging and disease^140–142^.

We specifically tested two gait parameters—spatial (Stride length) and temporal (Stride time)—to verify the presence of pink noise structure characteristic of a healthy, adaptable gait. We assessed the strength of long-range correlations in the time series of stride length and stride time following the Bayesian approach developed by Tyralis and Koutsoyiannis^143^. We chose this method because it performs well on short time series, as compared to more common methods like detrended fluctuation analysis^144,145^, which requires much longer time series data, and yields *H*_*fGn*_ comparable to the more commonly used detrended fluctuation analysis^146^.

Figure 6b,c represents violin plots of *H*_*fGn*_ values for the time series of Stride length and Stride time, respectively. The obtained *H*_*fGn*_ values indicate the pink noise structure in stride length and stride time, both of which are characteristic of a healthy gait^40,147–150^. Shuffling the original series yields *H*_*fGn*_ values resembling white noise, further confirming long-range correlations in the time series of Stride length and Stride time. Additionally, we observe high inter-individual variation in *H*_*fGn*_ values, accompanied by high intra-individual consistency in *H*_*fGn*_ values between the two sessions. This session-to-session test-retest reliability of the obtained *H*_*fGn*_ values points toward the uniqueness of individual gait patterns across both sessions^111^.

### Strengths and limitations

The present dataset has several strengths that make it unique among existing gait datasets. First, the present dataset reproduces many characteristics established in the literature on healthy human gait variability. Second, the application of several nonlinear analytical methods require long time series that span several thousand samples. An unusually long series of each of these variables (>40,000 samples for continuous variables and >2,000 samples for discrete variables, as opposed to ~ 2000 and ~ 200 samples, respectively, typical of existing datasets) makes the present dataset amenable to nonlinear analyses of gait variability which would be impractical with short series typical of existing datasets. Third, multiple recordings (*n* = 18) for each subject imply that the present dataset will significantly aid the modeling efforts to assess the uniqueness of individual gait patterns. In other words, the present database will aid the exploration of whether each person has a distinct “gaitprint” (i.e., gait characteristics) similar to how each person has a distinct fingerprint. If individuals have a unique gaitprint, then they should show consistency in gaitprint-defining statistical features across multiple recordings and sessions. This way, the present database could produce fundamental knowledge critical for predicting disease, predicting physiological declines, and improving rehabilitation.

The present dataset also has one fundamental weakness: the motion capture system recording the subjects’ motion. The sensors use the earth’s magnetic field to detect motions in the horizontal plane; hence, small variations in the magnetic field lead to drift; “an unwanted sliding movement on the horizontal plane of the complete biomechanical model in the motion capturing software”^151^. Furthermore, metal and magnetic objects in the measurement space can also alter this magnetic field, leading to phantom changes in direction. However, no vertical drift is observed, as no external factors aid the calculations in the vertical plane. Although the drift associated with the Noraxon Ultium Motion^TM^ is much smaller than observed while using the previous generations of inertial sensors^152,153^, a robust drift correction algorithm is recommended to enhance data accuracy. Drift correction is an area of ongoing development^154–157^, and we recommend implementing whatever state-of-the-art drift correction algorithm is available when analyzing the present dataset. However, we strongly believe that this weakness does not undermine the purported utility of the present dataset, as we have already shown the validity of the present dataset using several linear and nonlinear measures characteristic of healthy human gait variability.

## Usage Notes

We provide the present dataset as zipped folders in the data repository and also provide the scripts supporting analyses in MATLAB, Python, and R working environments; existing MATLAB and Python open-source software packages focused on gait could be used to analyze the raw data. For example, GaitPy is a Python library that provides functions to read and estimate the clinical characteristics of gait from accelerometer data (https://pypi.org/project/gaitpy/). Likewise, the Kinematics and Inverse Dynamics toolbox for MATLAB (https://www.mathworks.com/matlabcentral/fileexchange/58021-3d-kinematics-and-inverse-dynamics) provides functions for analyzing joint kinematics and dynamics. Other open-source software packages like biomechZoo^158^ could also be used to process, analyze, and visualize the present dataset. MATLAB and Python functions needed to perform nonlinear time series analyses on foot placement variabilities—such as those presented in the “Technical validation” section—can be obtained from the GitHub repository of the Nonlinear Analysis Core at the University of Nebraska Omaha (https://github.com/Nonlinear-Analysis-Core/NONANLibrary).

To facilitate the use of these software packages, the present dataset includes a MATLAB script entitled GaitPrint.m, which contains the function that computes all the spatiotemporal variables.

Finally, as part of an ongoing project, we plan to make publicly available gait datasets on five other populations:

- Group 2: Healthy middle-aged adults (36–55 years old)
- Group 3: Healthy older-aged adults (56 + years old)
- Group 4: Lower-limb amputees
- Group 5: Post-stroke patients
- Group 6: Patients with peripheral arterial disease (PAD)

Together these gait datasets will support and standardize the decision of researchers, clinicians, and therapists when assessing gait abnormalities or tracking the outcomes of rehabilitation interventions in older adults and clinical populations. They will also provide the reference database necessary for investigating whether healthy adults show unique individual gait patterns and what happens to these unique gait patterns with frailty associated with aging and disease. Users of our work will notice that the currently available dataset includes nonconsecutive subject IDs because our six groups were recruited and collected simultaneously. The inclusion of Groups 2–6 are to be released in future work.

### Supplementary information

Table 1

## Data Availability

The Matlab code GaitPrint.m, template scripts (MATLAB, Python, R), and all associated functions used for post-processing of all raw data are available as part of the database on figshare.

## References

[1] Phillips PJ, Sarkar S, Robledo I, Grother P, Bowyer K (2002). The gait identification challenge problem: data sets and baseline algorithm. International Conference on Pattern Recognition.

[2] Semwal VB, Raj M, Nandi GC (2015). Biometric gait identification based on a multilayer perceptron. Rob. Auton. Syst..

[3] Thang, H. M., Viet, V. Q., Thuc, N. D. & Choi, D. Gait identification using accelerometer on mobile phone. in *International Conference on Control, Automation and Information Sciences (ICCAIS)* 344–348. 10.1109/ICCAIS.2012.6466615 (2012).

[4] Cao P, Xia W, Ye M, Zhang J, Zhou J (2018). Radar-ID: Human identification based on radar micro-Doppler signatures using deep convolutional neural networks. IET Radar, Sonar Navig..

[5] Weich C, Vieten M (2020). M. The gaitprint: Identifying individuals by their running style. Sensors.

[6] Kumar P (2019). Multimodal gait recognition with inertial sensor data and video using evolutionary algorithm. IEEE Trans. Fuzzy Syst..

[7] Ariyanto, G. & Nixon, M. S. Model-based 3D gait biometrics. in *International Joint Conference on Biometrics (IJCB)* 1–7. 10.1109/IJCB.2011.6117582 (2011).

[8] Zhang Y, Huang Y, Wang L, Yu S (2019). A comprehensive study on gait biometrics using a joint CNN-based method. Pattern Recognit..

[9] Trentzsch K (2021). Using machine learning algorithms for identifying gait parameters suitable to evaluate subtle changes in gait in people with multiple sclerosis. Brain Sci..

[10] Jain AK, Prabhakar S, Pankanti S (2002). On the similarity of identical twin fingerprints. Pattern Recognit..

[11] Wiles, T. M., Kim, S. K., Stergiou, N. & Likens, A. D. Biomechanics using full body human movement variability gait data. *American Society of Biomechanics Annual Conference, Knoxville, TN*, (2023).

[12] Gabell A, Nayak USL (1984). The effect of age on variability in gait. J. Gerontol..

[13] Rosano C, Brach J, Studenski S, Longstreth WT, Newman AB (2007). Gait variability is associated with subclinical Brain vascular abnormalities in high-functioning older adults. Neuroepidemiology.

[14] Gierałtowski J, Żebrowski JJ, Baranowski R (2012). Multiscale multifractal analysis of heart rate variability recordings with a large number of occurrences of arrhythmia. Phys. Rev. E.

[15] Peng C-K (1995). Fractal mechanisms and heart rate dynamics: Long-range correlations and their breakdown with disease. J. Electrocardiol..

[16] Yamamoto Y (1995). On the fractal nature of heart rate variability in humans: Effects of vagal blockade. Am. J. Physiol. Integr. Comp. Physiol..

[17] Zhu P (2014). The relationship of retinal vessel diameters and fractal dimensions with blood pressure and cardiovascular risk factors. PLoS One.

[18] Soehle M, Czosnyka M, Chatfield DA, Hoeft A, Peña A (2007). Variability and fractal analysis of middle cerebral artery blood flow velocity and arterial blood pressure in subarachnoid hemorrhage. J. Cereb. Blood Flow Metab..

[19] Hu K, Lo M-T, Peng C-K, Liu Y, Novak V (2012). A nonlinear dynamic approach reveals a Long-term stroke effect on cerebral blood flow regulation at multiple time scales. PLOS Comput. Biol..

[20] Zappasodi F (2014). Fractal dimension of EEG activity senses neuronal impairment in acute stroke. PLoS One.

[21] Sharma M, Pachori RB (2017). & Rajendra Acharya, U. A new approach to characterize epileptic seizures using analytic time-frequency flexible wavelet transform and fractal dimension. Pattern Recognit. Lett..

[22] Li X (2005). Fractal spectral analysis of pre-epileptic seizures in terms of criticality. J. Neural Eng..

[23] Vaillancourt DE, Newell KM (2002). Changing complexity in human behavior and physiology through aging and disease. Neurobiol. Aging.

[24] Goldberger AL, Peng C-K, Lipsitz LA (2002). What is physiologic complexity and how does it change with aging and disease?. Neurobiol. Aging.

[25] Goldberger AL (2002). Fractal dynamics in physiology: Alterations with disease and aging. Proc. Natl. Acad. Sci..

[26] Stergiou N, Kent JA, McGrath D (2016). Human movement variability and aging. Kinesiol. Rev..

[27] Stergiou N, Decker LM (2011). Human movement variability, nonlinear dynamics, and pathology: Is there a connection?. Hum. Mov. Sci..

[28] Stergiou N, Harbourne RT, Cavanaugh JT (2006). Optimal movement variability: A new theoretical perspective for neurologic physical therapy. J. Neurol. Phys. Ther..

[29] Lockhart T, Stergiou N (2013). New perspectives in human movement variability. Ann. Biomed. Eng..

[30] Stergiou, N. *Nonlinear Analysis for Human Movement Variability*. (CRC Press, 2018).

[31] Stergiou, N. *Biomechanics and Gait Analysis*. (Academic Press, 2020).

[32] Eke A, Herman P, Kocsis L, Kozak LR (2002). Fractal characterization of complexity in temporal physiological signals. Physiol. Meas..

[33] Liebovitch LS, Yang W (1997). Transition from persistent to antipersistent correlation in biological systems. Phys. Rev. E.

[34] Hausdorff JM (1996). Fractal dynamics of human gait: Stability of long-range correlations in stride interval fluctuations. J. Appl. Physiol..

[35] Scafetta N, Griffin L, West BJ (2003). Hölder exponent spectra for human gait. Phys. A Stat. Mech. its Appl..

[36] Hausdorff JM, Peng CK, Ladin Z, Wei JY, Goldberger AL (1995). Is walking a random walk? Evidence for long-range correlations in stride interval of human gait. J. Appl. Physiol..

[37] Hausdorff JM (2007). Gait dynamics, fractals and falls: Finding meaning in the stride-to-stride fluctuations of human walking. Hum. Mov. Sci..

[38] Hausdorff JM (2009). Gait dynamics in Parkinson’s disease: Common and distinct behavior among stride length, gait variability, and fractal-like scaling. Chaos An Interdiscip. J. Nonlinear Sci..

[39] Herman T, Giladi N, Gurevich T, Hausdorff JM (2005). Gait instability and fractal dynamics of older adults with a “cautious” gait: Why do certain older adults walk fearfully?. Gait Posture.

[40] Hausdorff JM (1997). Altered fractal dynamics of gait: Reduced stride-interval correlations with aging and Huntington’s disease. J. Appl. Physiol..

[41] Palermo M, Lopes JM, André J, Cerqueira J, Santos C (2021). PhysioNet.

[42] Hicheur H, Vieilledent S, Berthoz A (2005). Head motion in humans alternating between straight and curved walking path: Combination of stabilizing and anticipatory orienting mechanisms. Neurosci. Lett..

[43] Fujii K, Kobayashi M, Sato M, Asakawa Y (2018). Relationship between straight and curved walking abilities among inpatients in the subacute phase according to walking independence level. *Phys. Ther*. Rehabil. Sci..

[44] Belluscio V (2020). Does curved walking sharpen the assessment of gait disorders? An instrumented approach based on wearable inertial sensors. Sensors.

[45] Courtine G, Schieppati M (2003). Human walking along a curved path. I. Body trajectory, segment orientation and the effect of vision. Eur. J. Neurosci..

[46] Courtine G, Schieppati M (2003). Human walking along a curved path. II. Gait features and EMG patterns. Eur. J. Neurosci..

[47] Warren WH, Kay BA, Zosh WD, Duchon AP, Sahuc S (2001). Optic flow is used to control human walking. Nat. Neurosci..

[48] Salinas MM, Wilken JM, Dingwell JB (2017). How humans use visual optic flow to regulate stepping during walking. Gait Posture.

[49] Watt JR (2010). A three-dimensional kinematic and kinetic comparison of overground and treadmill walking in healthy elderly subjects. Clin. Biomech..

[50] Alton F, Baldey L, Caplan S, Morrissey MC (1998). A kinematic comparison of overground and treadmill walking. Clin. Biomech..

[51] Lee SJ, Hidler J (2008). Biomechanics of overground vs. treadmill walking in healthy individuals. J. Appl. Physiol..

[52] Riley PO, Paolini G, Della Croce U, Paylo KW, Kerrigan DC (2007). A kinematic and kinetic comparison of overground and treadmill walking in healthy subjects. Gait Posture.

[53] Dingwell JB, Cusumano JP, Cavanagh PR, Sternad D (2000). Local dynamic stability versus kinematic variability of continuous overground and treadmill walking. J. Biomech. Eng..

[54] Hollman JH (2016). A comparison of variability in spatiotemporal gait parameters between treadmill and overground walking conditions. Gait Posture.

[55] Beauchet O, Launay CP, Annweiler C, Allali G (2015). Hippocampal volume, early cognitive decline and gait variability: Which association?. Exp. Gerontol..

[56] Brach JS, Berlin JE, VanSwearingen JM, Newman AB, Studenski SA (2005). Too much or too little step width variability is associated with a fall history in older persons who walk at or near normal gait speed. J. Neuroeng. Rehabil..

[57] Brach JS, Berthold R, Craik R, VanSwearingen JM, Newman AB (2001). Gait variability in community-dwelling older adults. J. Am. Geriatr. Soc..

[58] Maki BE (1997). Gait changes in older adults: Predictors of falls or indicators of fear?. J. Am. Geriatr. Soc..

[59] Nordin E, Moe-Nilssen R, Ramnemark A, Lundin-Olsson L (2010). Changes in step-width during dual-task walking predicts falls. Gait Posture.

[60] Owings TM, Grabiner MD (2004). Variability of step kinematics in young and older adults. Gait Posture.

[61] Owings TM, Grabiner MD (2004). Step width variability, but not step length variability or step time variability, discriminates gait of healthy young and older adults during treadmill locomotion. J. Biomech..

[62] Svoboda Z (2017). Variability of spatial temporal gait parameters and center of pressure displacements during gait in elderly fallers and nonfallers: A 6-month prospective study. PLoS One.

[63] Kastavelis, D., Mukherjee, M., Decker, L. M. & Stergiou, N. The effect of virtual reality on gait variability. (2010). 20587300

[64] Skiadopoulos A, Moore EE, Sayles HR, Schmid KK, Stergiou N (2020). Step width variability as a discriminator of age-related gait changes. J. Neuroeng. Rehabil..

[65] Hollman JH, McDade EM, Petersen RC (2011). Normative spatiotemporal gait parameters in older adults. Gait Posture.

[66] Stolze H, Kuhtz-Buschbeck JP, Mondwurf C, Jöhnk K, Friege L (1998). Retest reliability of spatiotemporal gait parameters in children and adults. Gait Posture.

[67] Reed LF, Urry SR, Wearing SC (2013). Reliability of spatiotemporal and kinetic gait parameters determined by a new instrumented treadmill system. BMC Musculoskelet. Disord..

[68] Herssens N (2018). Do spatiotemporal parameters and gait variability differ across the lifespan of healthy adults? A systematic review. Gait Posture.

[69] Kadaba MP (1989). Repeatability of kinematic, kinetic, and electromyographic data in normal adult gait. J. Orthop. Res..

[70] Boonstra AM, Fidler V, Eisma WH (1993). Walking speed of normal subjects and amputees: Aspects of validity of gait analysis. Prosthet. Orthot. Int..

[71] Winter DA (1984). Kinematic and kinetic patterns in human gait: Variability and compensating effects. Hum. Mov. Sci..

[72] Hussain R, Marmar Z (2021). Gait dataset of 14 Syrian above-knee amputees and 20 healthy subjects. Data Br..

[73] Moreira L, Figueiredo J, Fonseca P, Vilas-Boas JP, Santos CP (2021). Lower limb kinematic, kinetic, and EMG data from young healthy humans during walking at controlled speeds. Sci. Data.

[74] Reznick E (2021). Lower-limb kinematics and kinetics during continuously varying human locomotion. Sci. Data.

[75] Losing V, Hasenjäger M (2022). A multi-modal gait database of natural everyday-walk in an urban environment. Sci. Data.

[76] van der Zee TJ, Mundinger EM, Kuo AD (2022). A biomechanics dataset of healthy human walking at various speeds, step lengths and step widths. Sci. Data.

[77] Sharma A (2023). A non-laboratory gait dataset of full body kinematics and egocentric vision. Sci. Data.

[78] Ngo TT, Makihara Y, Nagahara H, Mukaigawa Y, Yagi Y (2014). The largest inertial sensor-based gait database and performance evaluation of gait-based personal authentication. Pattern Recognit..

[79] Moore JK, Hnat SK, van den Bogert AJ (2015). An elaborate data set on human gait and the effect of mechanical perturbations. PeerJ.

[80] Khandelwal S, Wickström N (2017). Evaluation of the performance of accelerometer-based gait event detection algorithms in different real-world scenarios using the MAREA gait database. Gait Posture.

[81] Schreiber C, Moissenet F (2019). A multimodal dataset of human gait at different walking speeds established on injury-free adult participants. Sci. Data.

[82] Luo Y (2020). A database of human gait performance on irregular and uneven surfaces collected by wearable sensors. Sci. Data.

[83] Pierleoni P, Pinti F, Belli A, Palma L (2020). A dataset for wearable sensors validation in gait analysis. Data Br..

[84] Bahadori S, Williams JM, Wainwright TW (2021). Lower limb kinematic, kinetic and spatial-temporal gait data for healthy adults using a self-paced treadmill. Data Br..

[85] Bertaux A (2022). Gait analysis dataset of healthy volunteers and patients before and 6 months after total hip arthroplasty. Sci. Data.

[86] Ravi DK (2020). Assessing the temporal organization of walking variability: A systematic review and consensus guidelines on detrended fluctuation analysis. Front. Physiol..

[87] Cohen, J. *Statistical Power Analysis for the Behavioral Sciences*. (Routledge, 2013).

[88] Cottam DS (2022). Measurement of uni-planar and sport specific trunk motion using magneto-inertial measurement units: The concurrent validity of Noraxon and Xsens systems relative to a retro-reflective system. Gait Posture.

[89] Berner K, Cockcroft J, Morris LD, Louw Q (2020). Concurrent validity and within-session reliability of gait kinematics measured using an inertial motion capture system with repeated calibration. J. Bodyw. Mov. Ther..

[90] Mundt M (2019). Assessment of the measurement accuracy of inertial sensors during different tasks of daily living. J. Biomech..

[91] Park S, Yoon S (2021). Validity evaluation of an inertial measurement Unit (IMU) In gait analysis using statistical parametric mapping (SPM). Sensors.

[92] Donaldson B, Bayne H, Bezodis NE (2021). Within-subject repeatability and between-subject variability in posture during calibration of an inertial measurement unit system. *ISBS Proc*. Arch..

[93] Wiles TM (2023). figshare.

[94] Beek PJ, Beek WJ (1988). Tools for constructing dynamical models of rhythmic movement. Hum. Mov. Sci..

[95] Levin, S. A. *Dynamical System Theory in Biology. Vol. 1. Stability Theory and Its Applications*. (Wiley, 1972).

[96] Stergiou N, Jensen JL, Bates BT, Scholten SD, Tzetzis G (2001). A dynamical systems investigation of lower extremity coordination during running over obstacles. Clin. Biomech..

[97] Stergiou N, Scholten SD, Jensen JL, Blanke D (2001). Intralimb coordination following obstacle clearance during running: The effect of obstacle height. Gait Posture.

[98] Likens, A. D. & Stergiou, N. Coordination and control: A dynamical systems approach to the analysis of human gait. in *Biomechanics and Gait Analysis* (ed. Stergiou, N.) 287–311 (Academic Press, 2020).

[99] Lamb PF, Stöckl M (2014). On the use of continuous relative phase: Review of current approaches and outline for a new standard. Clin. Biomech..

[100] Swinnen SP (2002). Intermanual coordination: From behavioural principles to neural-network interactions. Nat. Rev. Neurosci..

[101] Miller RH, Chang R, Baird JL, Van Emmerik REA, Hamill J (2010). Variability in kinematic coupling assessed by vector coding and continuous relative phase. J. Biomech..

[102] Haddad JM, van Emmerik REA, Wheat JS, Hamill J, Snapp-Childs W (2010). Relative phase coordination analysis in the assessment of dynamic gait symmetry. J. Appl. Biomech..

[103] Donker SF, Beek PJ (2002). Interlimb coordination in prosthetic walking: Effects of asymmetry and walking velocity. Acta Psychol. (Amst)..

[104] Ghanavati T (2014). Intra-limb coordination while walking is affected by cognitive load and walking speed. J. Biomech..

[105] Clark JE, Phillips SJ (1993). A longitudinal study of intralimb coordination in the first year of independent walking: A dynamical systems analysis. Child Dev..

[106] Byrne JE (2002). Comparison of gait patterns between young and elderly women: An examination of coordination. Percept. Mot. Skills.

[107] Yi LC, Sartor CD, Souza FT, Sacco ICN (2016). Intralimb coordination patterns in absent, mild, and severe stages of diabetic neuropathy: Looking beyond kinematic analysis of gait cycle. PLoS One.

[108] Hein T (2012). Using the variability of continuous relative phase as a measure to discriminate between healthy and injured runners. Hum. Mov. Sci..

[109] Armitano CN, Morrison S, Russell DM (2018). Coordination stability between the legs is reduced after anterior cruciate ligament reconstruction. Clin. Biomech..

[110] Kurz MJ, Stergiou N, Buzzi UH, Georgoulis AD (2005). The effect of anterior cruciate ligament recontruction on lower extremity relative phase dynamics during walking and running. Knee Surgery, Sport. Traumatol. Arthrosc..

[111] Raffalt, P. C. *et al*. Day-to-day reliability of nonlinear methods to assess walking dynamics. *J. Biomech. Eng*. **140**, (2018). 10.1115/1.4041044 30264156

[112] Wolf A, Swift JB, Swinney HL, Vastano JA (1985). Determining Lyapunov exponents from a time series. Phys. D Nonlinear Phenom..

[113] Rosenstein MT, Collins JJ, De Luca CJ (1993). A practical method for calculating largest Lyapunov exponents from small data sets. Phys. D Nonlinear Phenom..

[114] Buzzi UH, Stergiou N, Kurz MJ, Hageman PA, Heidel J (2003). Nonlinear dynamics indicates aging affects variability during gait. Clin. Biomech..

[115] Cavanaugh, J. T. & Stergiou, N. Gait variability: A theoretical framework for gait analysis and biomechanics. in *Biomechanics and Gait Analysis* (ed. Stergiou, N.) 251–286 (Academic Press, 2020).

[116] Dingwell JB, Cusumano JP (2000). Nonlinear time series analysis of normal and pathological human walking. *Chaos An Interdiscip*. J. Nonlinear Sci..

[117] Myers SA (2009). Gait variability is altered in patients with peripheral arterial disease. J. Vasc. Surg..

[118] Huisinga JM, Mancini M, St. George RJ, Horak FB (2013). Accelerometry reveals differences in gait variability between patients with multiple sclerosis and healthy controls. Ann. Biomed. Eng..

[119] Myers SA, Stergiou N, Pipinos II, Johanning JM (2010). Gait variability patterns are altered in healthy young individuals during the acute reperfusion phase of ischemia-reperfusion. J. Surg. Res..

[120] Rahman H, Pipinos II, Johanning JM, Myers SA (2021). Gait variability is affected more by peripheral artery disease than by vascular occlusion. PLoS One.

[121] IJmker T, Lamoth CJC (2012). Gait and cognition: The relationship between gait stability and variability with executive function in persons with and without dementia. Gait Posture.

[122] Piórek M, Josiński H, Michalczuk A, Świtoński A, Szczȩsna A (2017). Quaternions and joint angles in an analysis of local stability of gait for different variants of walking speed and treadmill slope. Inf. Sci. (Ny)..

[123] Mehdizadeh S (2018). The largest Lyapunov exponent of gait in young and elderly individuals: A systematic review. Gait Posture.

[124] England SA, Granata KP (2007). The influence of gait speed on local dynamic stability of walking. Gait Posture.

[125] Qian, Y., Yang, K., Zhu, Y., Wang, W. & Wan, C. Local dynamic stability of self-paced treadmill walking versus fixed-speed treadmill walking. *J. Biomech. Eng*. **142**, (2020). 10.1115/1.4045595 31802107

[126] Stergiou N, Yu Y, Kyvelidou A (2013). A perspective on human movement variability with applications in infancy motor development. Kinesiol. Rev..

[127] Kello CT, Anderson GG, Holden JG, Van Orden GC (2008). The pervasiveness of 1/f scaling in speech reflects the metastable basis of cognition. Cogn. Sci..

[128] Kello CT, Bella SD, Médé B, Balasubramaniam R (2017). Hierarchical temporal structure in music, speech and animal vocalizations: Jazz is like a conversation, humpbacks sing like hermit thrushes. J. R. Soc. Interface.

[129] Van Orden, G. C., Kloos, H. & Wallot, S. Living in the pink: Intentionality, wellbeing, and complexity. in *Handbook of the Philosophy of Science* (ed. Hooker, C.) **vol. 10** 629–672 (Elsevier, 2011).

[130] Delignières D, Marmelat V (2014). Strong anticipation and long-range cross-correlation: Application of detrended cross-correlation analysis to human behavioral data. Phys. A Stat. Mech. its Appl..

[131] Diniz A (2011). Contemporary theories of 1/*f* noise in motor control. Hum. Mov. Sci..

[132] Likens, A. D. & Stergiou, N. A tutorial on fractal analysis of human movements. in *Biomechanics and Gait Analysis* (ed. Stergiou, N.) 313–344 (Academic Press, 2022).

[133] Gilden DL (2001). Cognitive emissions of 1/*f* noise. Psychol. Rev..

[134] Van Orden GC, Holden JG, Turvey MT (2003). Self-organization of cognitive performance. J. Exp. Psychol. Gen..

[135] Jordan K, Challis JH, Cusumano JP, Newell KM (2009). Stability and the time-dependent structure of gait variability in walking and running. Hum. Mov. Sci..

[136] Jordan K, Challis JH, Newell KM (2006). Long range correlations in the stride interval of running. Gait Posture.

[137] Lindsay TR, Noakes TD, McGregor SJ (2014). Effect of treadmill versus overground running on the structure of variability of stride timing. Percept. Mot. Skills.

[138] Jordan K, Challis JH, Newell KM (2007). Speed influences on the scaling behavior of gait cycle fluctuations during treadmill running. Hum. Mov. Sci..

[139] Terrier P, Dériaz O (2011). Kinematic variability, fractal dynamics and local dynamic stability of treadmill walking. J. Neuroeng. Rehabil..

[140] Kaipust JP, McGrath D, Mukherjee M, Stergiou N (2013). Gait variability is altered in older adults when listening to auditory stimuli with differing temporal structures. Ann. Biomed. Eng..

[141] Vaz JR, Knarr BA, Stergiou N (2020). Gait complexity is acutely restored in older adults when walking to a fractal-like visual stimulus. Hum. Mov. Sci..

[142] Marmelat V, Meidinger RL (2019). Fractal analysis of gait in people with Parkinson’s disease: Three minutes is not enough. Gait Posture.

[143] Tyralis H, Koutsoyiannis D (2014). A Bayesian statistical model for deriving the predictive distribution of hydroclimatic variables. Clim. Dyn..

[144] Peng C-K (1994). Mosaic organization of DNA nucleotides. Phys. Rev. E.

[145] Peng C-K, Havlin S, Stanley HE, Goldberger AL (1995). Quantification of scaling exponents and crossover phenomena in nonstationary heartbeat time series. Chaos An Interdiscip. J. Nonlinear Sci..

[146] Likens, A. D., Mangalam, M., Wong, A. Y., Charles, A. C. & Mills, C. Better than DFA? A Bayesian method for estimating the Hurst exponent in behavioral sciences. *arXiv:2301.11262*.

[147] Mangalam M, Kelty-Stephen DG, Sommerfeld JH, Stergiou N, Likens AD (2023). Temporal organization of stride-to-stride variations contradicts predictive models for sensorimotor control of footfalls during walking. PLoS One.

[148] Mangalam M (2023). Leveraging a virtual alley with continuously varying width modulates step width variability during self-paced treadmill walking. Neurosci. Lett..

[149] Raffalt PC, Sommerfeld JH, Stergiou N, Likens AD (2023). Stride-to-stride time intervals are independently affected by the temporal pattern and probability distribution of visual cues. Neurosci. Lett..

[150] Raffalt PC, Stergiou N, Sommerfeld JH, Likens AD (2021). The temporal pattern and the probability distribution of visual cueing can alter the structure of stride-to-stride variability. Neurosci. Lett..

[151] Damgrave, R. G. J. & Lutters, D. The drift of the Xsens moven motion capturing suit during common movements in a working environment. in *Proceedings of the 19th CIRP Design Conference–Competitive Design* (Cranfield University Press, 2009).

[152] Dejnabadi H, Jolles BM, Casanova E, Fua P, Aminian K (2006). Estimation and visualization of sagittal kinematics of lower limbs orientation using body-fixed sensors. IEEE Trans. Biomed. Eng..

[153] Luinge HJ, Veltink PH (2005). Measuring orientation of human body segments using miniature gyroscopes and accelerometers. Med. Biol. Eng. Comput..

[154] Butt, H. T. *et al*. Inertial motion capture using adaptive sensor fusion and joint angle drift correction. in *22th International Conference on Information Fusion (FUSION)* 1–8. 10.23919/FUSION43075.2019.9011359 (2019).

[155] Munoz Diaz E, Caamano M, Sánchez FJ (2017). Landmark-based drift compensation algorithm for inertial pedestrian navigation. Sensors.

[156] Wittmann F, Lambercy O, Gassert R (2019). Magnetometer-based drift correction during rest in imu arm motion tracking. Sensors.

[157] Dai, Z., Lu, C. & Jing, L. Time drift compensation method on multiple wireless motion capture nodes. in *13th International Conference on Human System Interaction (HSI)* 266–271. 10.1109/HSI49210.2020.9142648 (2020).

[158] Dixon PC, Loh JJ, Michaud-Paquette Y, Pearsall D (2017). J. biomechZoo: An open-source toolbox for the processing, analysis, and visualization of biomechanical movement data. Comput. Methods Programs Biomed..

