# Supplementary material for: NONAN GaitPrint: An IMU gait database of healthy young adults
Source: Sci Data. 2023 Dec 5;10:867. doi: 10.1038/s41597-023-02704-z (PMC10698035; doi:10.1038/s41597-023-02704-z)
Supplement: Supplementary file 1 — Table 1 [file 41597_2023_2704_MOESM1_ESM.docx]

**Supplementary Table 1.** A representative sample of existing gait datasets (all datasets are publicly available online and can be accessed via links provided in the respective publication pages).

| **Year** | **Gait dataset** | **No of subjects** | **No of sequences** | **Walking environment** | **Approach** | **Parameters** | **Motion capture technology** | **Application** |
| --- | --- | --- | --- | --- | --- | --- | --- | --- |
| 2014 | Ngo et al.^78^ | 744 | Slope (flat, up, and down) | Overground | 4 IMUs, waist | Slope | IMUs | Gait recognition |
| 2015 | Moore et al.^79^ | 15 | 1 × 120-s Self-paced walking vs. 1 × 480-s Perturbation | Instrumented treadmill | 47 reflective markers on the whole body | No-perturbation vs. Perturbation | Infrared motion capture | Estimation of gait parameters during perturbations |
| 2017 | Khandelwal & Wickström^80^ | 20 | Variable duration trial; Variable speeds × Variable inclination | Treadmill; Overground | 4 IMUs, left hand, waist, and feet | Surface (treadmill, overground); Mode (walking, running) | IMUs | Gait event detection |
| 2019 | Schreiber & Moissenet^81^ | 50 | 3 × 10-m trials; 5 Speeds (0–0.4 m/s, 0.4–0.8 m/s, 0.8–1.2 m/s, self-selected spontaneous, and fast speeds) | Gait lab, 10-m walkway | 52 reflective markers on the whole body | Speed | Infrared motion capture | Estimation of gait parameters |
| 2020 | Luo et al.^82^ | 30 | 57 × 16.4±4.2-s trial; Grade (up-, down-, and cross-slopes), regularity (paved, uneven stone, grass), and stair negotiation (up and down) | Urban environment | 6 IMUs on the lower limbs | Terrain | IMUs | Gait recognition |
| 2020 | Pierleoni et al.^83^ | 5 | 10 × 11-m trials | Gait lab, 10-m walkway | 2 IMUs + 8 reflective markers, on the feet | None | IMUs; Infrared motion capture | Estimation of gait parameters |

**Supplementary Table 1 continued.**

| **Year** | **Gait dataset** | **No of subjects** | **No of sequences** | **Walking environment** | **Approach** | **Parameters** | **Motion capture technology** | **Application** |
| --- | --- | --- | --- | --- | --- | --- | --- | --- |
| 2021 | Bahadori et al.^84^ | 100 | 1 × 5-min trial; Self-paced | Instrumented treadmill | 25 reflective markers on the lower limbs | — | Infrared motion capture | Estimation of gait parameters |
| 2021 | Bertaux et al.^85^ | 80 | 10 × 6-m | Gait lab, 6-m walkway | 35 reflective markers on the whole body | — | Infrared motion capture | Estimation of gait parameters |
| 2021 | Hussain and Marmar^72^ | 20 | 3–5 Self-paced trails lasting the calibrated space | Gait lab | 22 reflective markers on the lower limbs | — | Infrared motion capture | Estimation of gait parameters |
| 2021 | Moreira et al.^73^. | 16 | 10 × 10-m trials; 7 Speeds (1.0, 1.5, 2.0, 2.5, 3.0, 3.5, and 4.0 km/h) | Gait lab, 10-m walkway | 24 reflective markers on the lower limbs | Speed | Infrared motion capture | Validate and enhance human biomechanical gait model; Provide reference trajectory for personalized control of robotic assistive devices |
| 2021 | Palermo et al.^41^ | 14 | 3 × unspecified length trials; 3 Speeds (0.3, 0.5, and 0.7 m/s) × 3 Tasks (walking forward, turning left, turning right) × 3 Trials × 3 Locations | Not specified | 17 IMUs on the whole body | Speed, Task, Location | IMUs | Vision-based pose estimation; Movement forecasting; Estimation of gait parameters |

**Supplementary Table 1 continued.**

| **Year** | **Gait dataset** | **No of subjects** | **No of sequences** | **Walking environment** | **Approach** | **Parameters** | **Motion capture technology** | **Application** |
| --- | --- | --- | --- | --- | --- | --- | --- | --- |
| 2021 | Reznick et al.^74^ | 10 | 1 × 30-s max trial; Walking at multiple Inclines (±0°, 5°, and 10°) and Speeds (0.8 m/s, 1 m/s, and 1.2 m/s);  Running at multiple Speeds (1.8 m/s; 2 m/s; 2.2 m/s, and 2.4 m/s); Walking and Running with Constant acceleration (±0.2 m/s^2^, 0.5 m/s^2^); Stair ascent/descent with multiple Inclines (20°, 25°, 30°, and 35°) | Instrumented treadmill and stairs | 24 reflective markers on the lower limbs | Mode (walking, running); Inclination; Speed;  Acceleration; Direction of stair climbing | Infrared motion capture | Estimation of gait parameters for robotic prostheses and exoskeletons |
| 2022 | Losing & Hasenjäger^75^ | 20 | 3 × <4-min trials | Urban environment | 17 IMUs on the whole body | Walking speed variation due to walking on variable terrain (level walking, walking up/down ramps and stairs) | IMUs | Analysis and prediction of gait patterns in a variable real-life environment; Gait recognition |
| 2022 | van der Zee^76^ | 10 | 33 × 60 s trials; Speed, Step length, and Step width | Treadmill | 37 reflective markers on the whole body | Variations of speed, step length, and step width | Infrared motion capture | Estimation of gait parameters |
| 2023 | Sharma et al.^77^ | 76 | Variable across subjects | Urban indoor environment | 17 IMUs on the whole body | Walking speed variation due to walking on variable terrain (level walking, walking up/down ramps and stairs) | IMUs | Analysis and prediction of gait patterns in a variable real-life environment; Gait recognition |

**References**

1. Palermo, M., Lopes, J. M., André, J., Cerqueira, J. & Santos, C. A multi-camera and multimodal dataset for posture and gait analysis (version 1.0.0). *PhysioNet* https://doi.org/10.13026/fyxw-n385 (2021).
2. Hussain, R. & Marmar, Z. Gait dataset of 14 Syrian above-knee amputees and 20 healthy subjects. *Data Br.* **38**, 107365 (2021).
3. Moreira,L.,Figueiredo,J.,Fonseca,P.,Vilas-Boas, J. P. & Santos, C. P. Lower-limb kinematic, kinetic, and EMG data from young healthy humans during walking at controlled speeds. *Sci. Data* **8**, 103 (2021).
4. Reznick, E. *et al*. Lower-limb kinematics and kinetics during continuously varying human locomotion. *Sci. Data* **8**, 282 (2021).
5. Losing, V. & Hasenjäger, M. A multi-modal gait database of natural everyday-walk in an urban environment. *Sci. Data* **9**, 473 (2022).
6. vanderZee, T. J., Mundinger, E. M. & Kuo, A. D. A biomechanics dataset of healthy human walking at various speeds, step lengths and step widths. *Sci. Data* **9**, 704 (2022).
7. Sharma, A. *et al*. A non-laboratory gait dataset of full body kinematics and egocentric vision. *Sci. Data* **10**, 26 (2023).
8. Ngo, T. T., Makihara, Y., Nagahara, H., Mukaigawa, Y. & Yagi, Y. The largest inertial sensor-based gait database and performance evaluation of gait-based personal authentication. *Pattern Recognit.* **47**, 228–237 (2014).
9. Moore, J. K., Hnat, S. K. & vanden Bogert, A. J. An elaborate dataset on human gait and the effect of mechanical perturbations. *PeerJ* **3**, e918 (2015).
10. Khandelwal, S. & Wickström, N. Evaluation of the performance of accelerometer-based gait event detection algorithms in different real-world scenarios using the MAREA gait database. *Gait Posture* **51**, 84–90 (2017).
11. Schreiber, C. & Moissenet, F. A multimodal dataset of human gait at different walking speeds established on injury-free adult participants. *Sci. Data* **6**, 111 (2019).
12. Luo, Y. *etal*. A database of human gait performance on irregular and uneven surfaces collected by wearable sensors. *Sci. Data* **7**, 219 (2020).
13. Pierleoni, P., Pinti, F., Belli, A. & Palma, L. A dataset for wearable sensors validation in gait analysis. *Data Br.* **31**, 105918 (2020).
14. Bahadori, S., Williams, J. M. & Wainwright, T. W. Lower limb kinematic, kinetic and spatial-temporal gait data for healthy adults using a self-paced treadmill. *Data Br.* **34**, 106613 (2021).
15. Bertaux, A. *et al*. Gait analysis dataset of healthy volunteers and patients before and 6 months after total hip arthroplasty. *Sci. Data* **9**, 399 (2022).
